# A Review on the Production and Characteristics of Cheese Powders

**DOI:** 10.3390/foods13142204

**Published:** 2024-07-12

**Authors:** Gaurav Kr Deshwal, F.N.U. Akshit, Ipek Altay, Thom Huppertz

**Affiliations:** 1 Department of Food Chemistry and Technology, Teagasc Food Research Centre, P61C996 Cork, Ireland; gaurav.deshwal@wur.nl; 2 Department of Agrotechnology and Food Sciences, Wageningen University, Bornse Weilanden 9, 6708 WG Wageningen, The Netherlands; 3 Dairy Technology Division, ICAR-National Dairy Research Institute, Karnal 132001, Haryana, India; 4 Department of Dairy and Food Science, South Dakota State University, Brookings, SD 57006, USA; 5 Research Group for Food Production Engineering, National Food Institute, Technical University of Denmark, Soeltofts Plads 227, 2800 Kongens Lyngby, Denmark; ipeal@food.dtu.dk; 6 FrieslandCampina, Stationsplein 4, 3818 LE Amersfoort, The Netherlands; 7 School of Food and Nutritional Sciences, University College Cork, T12YN60 Cork, Ireland

**Keywords:** calcium-sequestering salts, spray-drying, cheese flavor

## Abstract

Cheese powder is a product resulting from the removal of moisture from cheese. At first, cheese emulsion is prepared by dissolving cheese(s) with water and calcium sequestering salts followed by drying. The desirable characteristics of cheese powder are high solubility, no lumps, storage stability, and imparting a typical cheesy flavor to the final product. Many current studies on cheese powder are focused on reducing calcium-sequestering salts (CSSs) to reduce the sodium content of cheese powder. This review discusses the production processes and physio-chemical properties of cheese emulsions and powders, aiming to enhance current understanding and identifying potential research gaps. Furthermore, strategies for producing cheese powder without CSSs, including pH adjustment, homogenization, and addition of dairy components such as buttermilk powder and sodium caseinate, are elaborated upon. Processing variables such as heating conditions during the preparation of cheese emulsion may vary with the type and age of the cheese used and product formulation. These conditions also effect the characteristics of cheese powders. On the other hand, producing a stable cheese emulsion without CSSs is challenging due to impaired emulsification of fat. The combined use of buttermilk powder and sodium caseinate among various alternatives has shown promising results in producing cheese powder without CSSs. However, future research on replacing CSSs should focus on combining two or more strategies together to produce cheese powder without CSSs. The combination of pH adjustment and dairy ingredients and the use of novel processing technologies with different ingredients are interesting alternatives.

## 1. Introduction

The origin of dehydrated cheese products can be traced to World War II, when the US Army used industrially made cheese-based dried ingredients because of their ability to withstand higher storage temperatures than are used in the refrigerated storage of natural cheese [1,2]. Ever since then, dehydrated cheese products have become a significant dairy ingredient, mostly utilized in food applications as a flavoring ingredient and nutritional supplement. This is signified by the current value of the global market of cheese powder at USD 571 million in 2022, which is projected to reach USD 721 million in 2027, with a compound annual growth rate (CAGR) of 6% [3].

Key advantages of dehydrated cheese products over natural cheeses include longer shelf life, ease of transportation and storage, ease of use during food application by wet or dry blending, and enhanced and diverse flavor profiles due to different types of cheeses used in manufacturing [1,2]. Dehydrated cheese products can be divided into three different categories, namely dried grated cheese, natural and extended cheese powder, and enzyme-modified cheese (EMC) powder [1]. The preparation of dried grated cheese involves grinding hard cheese to fine particles followed by drying in a fluidized bed dryer using low-humidity air at an inlet air temperature of less than 30 °C or freeze-drying [1]. Natural cheese powder is prepared by blending natural cheeses and calcium-sequestering salts (CSSs), whereas extended cheese powder may also further contain other dairy and non-dairy ingredients such as skim milk solids, whey, lactose, starches, maltodextrin, and flavoring ingredients [2]. EMC powders are prepared by adding enzymes, primarily proteinases or lipases, to a cheese emulsion to develop an intense flavor similar to ripened cheese, which is subsequently dried by spray-drying [1]. One of the advantages of EMC powders over cheese powders is that the flavor intensity can be improved by about 15–30 times more than addition of natural cheeses [4]. In this review, we will focus primarily on natural and extended cheese powders prepared by spray-drying.

The process of preparing natural cheese powder begins with formulating a cheese emulsion consisting of the main ingredients: cheese, water, and CSSs. For extended cheese powders, other components, such as skim milk solids, whey, and flavor enhancers, can also be incorporated in the formulation depending on the requirement. The ingredients are then blended and heated to prepare the emulsion, which is dried into cheese powder [2]. It is crucial to achieve a stable, homogeneous, and easily-pumpable cheese emulsion for the production of cheese powder [5]. The stability of the cheese emulsion is influenced by cheese variety, the type and concentration of CSSs, processing conditions, dry matter, and pH of the emulsion [5]. CSSs play a key role in providing homogeneity and stability to cheese emulsions by chelating calcium from milk proteins, improving fat emulsification, and making the emulsion suitable for the drying process [6]. In recent times, there has been growing interest in searching for alternatives to CSSs in cheese emulsions, driven by the desire to reduce salt in foods [6,7,8]. In this respect, several studies have investigated the effect of pH adjustment and homogenization [6,9] as well as the addition of sweet whey powder [10], sodium caseinate, or buttermilk powder [5,11] on the properties of cheese powder. Similarly, the use of non-dairy ingredients like maltodextrin [12,13,14] and microparticulated whey protein in reduced-fat cheese emulsions and powders [15,16] has also been investigated.

Currently, a detailed review on the manufacture of cheese emulsion and powder is lacking. The present review is focused on existing knowledge of the preparation of cheese emulsion and cheese powder, the alternatives employed for emulsification, and the consequent impact on the properties of cheese powder. This will assist researchers in identifying potential research gaps and generating perspectives for future research studies focused on cheese powder.

## 2. Applications of Cheese Powder

While cheese powders serve as a multifunctional ingredient in various food applications contributing to texture, mouthfeel, or color, they are primarily used as a flavoring agent. Typically, the quantity of cheese powder utilized in food products ranges between 5 and 10%, varying according to the desired intensity of flavor [17]. Cheese powder is used as a versatile ingredient to offer a palatable burst of cheese flavor in snacks; as a binding agent in nut coatings; as a thickening agent in instant premixes, ready-to-eat meals, cheese sauces, and dips; as a leavening agent in bakery products; and to improve the texture, consistency, and shelf-life of seasoning blends [15,18,19]. Some reported applications of cheese powder in different food formulations are summarized in Table 1.

### 2.1. Cheese Powder as a Flavoring Agent

Cheese powder has gained widespread use in the food industry as a versatile flavoring agent in sauces, soups, savory dressings, baked foods, and coatings including popcorn, nachos, and tortilla shells [1,2,9]. Cheese powder is also used in snack foods to enhance their flavor [2]. Additionally, cheese powders have been combined with spices for creating seasonings or savory additives [1]. In addition to enhancing sensory attributes, the incorporation of cheese powder (10–20% *w*/*w*) into ready-to-eat rice and corn flour-based extruded snacks has been shown to increase their protein content [20]. Similarly, the addition of cheese powder during the freeze-drying of broccoli powder improved its color, flavor, and texture attributes while retaining higher levels of total polyphenols [21]. The addition of cheese powder at a 40–50% level to a black gram flour-based deep-fried snack improved its cheesy flavor and mouthfeel. However, browning was observed at higher processing temperatures (180 °C), which decreased the overall acceptability [22]. Hannon et al. [23] explored the use of up to 1% of EMC powder in the manufacture of ingredient-type Cheddar cheese to accelerate flavor development and the ripening process.

**Table 1:** Applications of cheese powder in different food products.

Food Products	Functionality	Reference
Rice and corn flour-based extruded snacks	Improved nutrient content and higher expansion of snacks	[20]
Broccoli sprout powder	Higher retention of polyphenols and carotenoids	[21]
Black gram flour-based deep-fried snack	Cheese flavor leading to higher acceptability	[22]
Dry fermented sausages	Increased sourness and cheesy flavor intensity at reduced salt content	[24]
Meat emulsion sausages	Increased hardness and meat flavor intensity	[25]
Mayonnaise	Good fat emulsification and development of gel-like structure	[26]

Generally, the flavor compounds in cheese powder are esters, aldehydes, sulfur compounds, alcohols, ketones, terpenes, and pyrazines [27]. For instance, Danbo cheese powder is characterized by sulfur compounds (dimethyl disulphide and dimethyl trisulphide) and alcohols (2-phenyl ethanol), whereas Emmental cheese powder is characterized by esters (ethyl acetate and propyl butanoate), methyl ketones (2- and 3-Methyl butanal) and aldehydes (hexanal) [27]. Similarly, Blue cheese powder is characterized by esters (methyl butanoate, pentanoate and hexanoate, etc.), aldehydes (butanal, 2-methylpropanal and 2-methylbutanal) and ketones (2-pentanone/diacetyl and hexanal), whereas smear-ripened cheese powder is characterized by sulfur compounds (dimethyl disulphide and dimethyl trisulphide) and phenols (indole and phenol) [28]. These flavor compounds have been linked to fruity, buttery, sharp, salty, smoky, pungent, sour, and umami tastes in different varieties of cheeses [29]. Overall, the flavor of cheese powder is to a large extent defined by the cheese type(s) used in the product.

The role of cheese powder as a flavor ingredient has also been explored in meat products. For instance, in dry fermented sausages, the addition of different cheese powders prepared from extra hard cheese; a mix of extra hard, blue, and smear cheese; and organic extra hard cheese at a 1% (*w*/*w*) level enhanced the flavor intensity and sourness [24]. Likewise, the addition of cheese powders at 2% (*w*/*w*) to meat emulsion sausages intensified the meat flavor. The addition of blue cheese powder increased the ketones, alcohols, and esters in the sausages, whereas brown cheese powder enhanced Maillard browning compounds and umami taste [25].

### 2.2. Cheese Powder as an Emulsifying Agent

Several studies have explored the use of cheese powder as a natural emulsifier due to the presence of caseins or polypeptides derived therefrom, which possess amphiphilic properties. For example, da Silva et al. [30] demonstrated that in the stabilization of oil-in-water emulsions, Camembert cheese powder outperformed sodium caseinate as an emulsifier. The emulsion stability increased with increasing protein content [30]. The use of cheese powder obtained from cheeses with different ripening times as an emulsifier for oil-in-water emulsions affected emulsion stability. A higher and faster instability of emulsions was observed for cheese powder made from 30- and 45-week-old cheese compared to 16-week-old cheese. This was attributed to increased protein degradation with increased ripening time [31]. The addition of Cheddar cheese powder (4%, *w*/*w*) in mayonnaise formulations resulted in similar rheological attributes as in mayonnaise with egg yolk as an emulsifier. Mayonnaise with Cheddar cheese powder showed effective fat emulsification but lacked water-binding ability [26]. However, mayonnaise made with Camembert cheese powder showed inferior rheological characteristics and emulsion stability, highlighting the influence of the type of cheese powder on its end application [26]. These findings emphasize the diverse applications and functionalities of cheese powder in the food industry.

## 3. Process for Production of Cheese Powder

**Figure 1:**
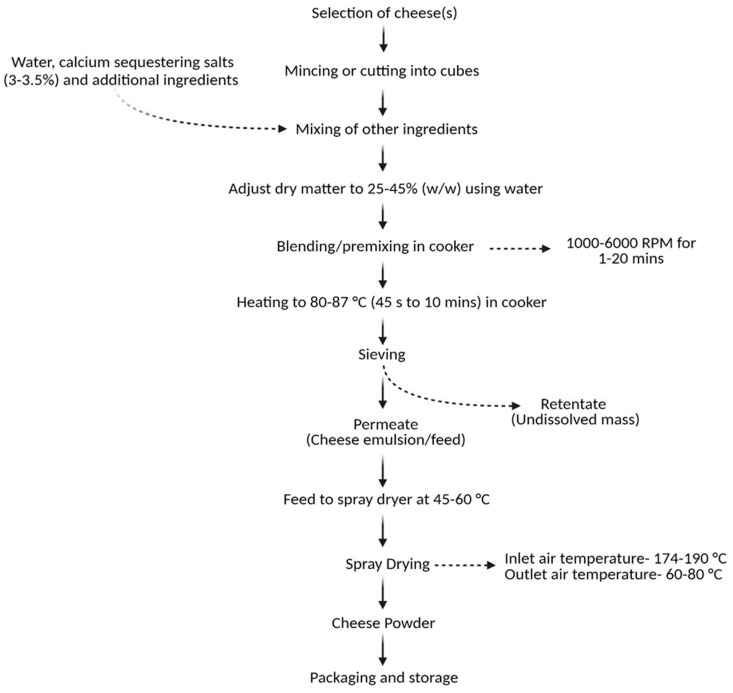
Process flow diagram for the production of cheese powder (created with Biorender.com).

To enable the spray-drying of cheese to cheese powder, it is first necessary to convert the cheese into the liquid form [32], referred to as a cheese emulsion. A process flow diagram for a typical production process of cheese powder is shown in Figure 1. Preparation of the cheese emulsion involves three main steps: (1) selection of cheese(s) and other ingredients; (2) comminuting the cheeses and mixing them with water and other ingredients; (3) heating this mixture to a specified temperature and holding for a desired time at a suitable mixing speed. The specific time and temperature levels for heating cheese emulsion vary across the literature (Table 2), for instance, 80 °C for 1 min [33], 85 °C for 45 s [5], 80 °C for 5 min [6], and 87 °C for 45 s [34]. Processing temperatures above 85 °C should be avoided if possible, since these can cause loss of volatile compounds [2]. The resulting hot mixture is passed through a sieve, and the filtrate is termed cheese feed, whereas the residual undissolved components of cheese are present in the retentate [7,9]. It is also common to include a homogenization step into cheese emulsion processing to improve colloidal stability before spray-drying. This process is usually conducted in two stages, applying pressures of, e.g., 15 and 5 MPa, respectively [1]. Finally, the cheese emulsion is pasteurized and spray-dried, which is discussed in Section 5.

## 4. Cheese Emulsion

**Figure 2:**
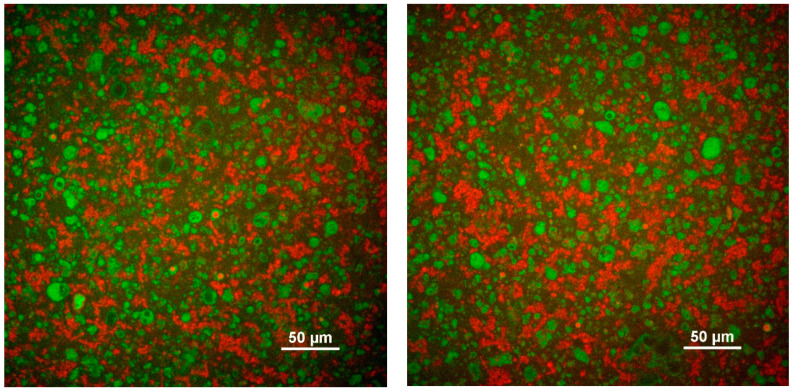
Confocal laser scanning microscopy images of cheese emulsions with different formulation. Green and red regions represent protein and fat, respectively.

The feed provided to the spray-dryer for the preparation of cheese powder is known as the cheese emulsion. Various types of cheeses, including Cheddar, Danbo, Camembert, Gouda, and Emmental, can be used in the preparation of cheese emulsions and cheese powders [10,11]. The process of preparing the cheese emulsion has many similarities to the manufacturing process of processed cheese, with the clear distinction that cheese emulsion has a lower dry matter content (25–45%) [5,9,13] and is maintained at elevated temperature (45–60 °C) prior to drying [36]. In Figure 2, confocal microscopy images of stable cheese emulsions produced on an industrial scale are presented. These cheese emulsions were prepared with different formulations, both containing CSSs. Both emulsions show relatively uniformly dispersed fat droplets surrounded by the protein aggregates. 

The influence of various compositional and processing factors on the properties of cheese emulsions are discussed in the following subsections, wherein studies have primarily focused on various approaches as an alternative to CSSs in cheese emulsion preparations.

### 4.1. Cheese Type

Various cheeses exhibit diverse physicochemical properties due to differences in their age, resulting in varying levels of protein and fat degradation within the matrix. For example, matured cheeses are usually preferable for enhancing the flavor of the final cheese powder, whereas young cheeses are used due to their high amount of intact casein, although they are more difficult to dissolve [2]. In addition, the use of matured cheese with a sharp flavor can compensate for the loss of volatile compounds during thermal processing [32]. These distinctions contribute to variations in the solubilization of caseins, thereby influencing the properties of cheese emulsion and powder. Therefore, different types of cheese have been utilized for the preparation of cheese emulsions to explore the potential of their conversion into cheese powder. A mixture of Cheddar and Camembert cheese was prepared with 5 g CSSs per 100 g (on dry matter basis) feed and produced a cheese emulsion without phase separation and sediment formation [36]. This stability was attributed to the higher pH and lower calcium concentration in Camembert and Cheddar cheese emulsions compared to Cheddar alone or when mixed with soft white cheese [36]. CSS-free Camembert cheese emulsions exhibited protein aggregates with large fat clusters, leading to a higher viscosity compared to CSS-free Cheddar cheese emulsions [7]. Both types of cheese emulsion were unstable and showed separation of oil and water phases. Cheddar cheese with a higher colloidal calcium content exhibited inferior solids recovery as CCP affected the disintegration of caseins and hindered emulsification. Camembert cheese protein had lower functionality due to higher proteolysis than in Cheddar cheese, lowering the emulsifying ability of proteins as they become more water-soluble with increased proteolysis [7].

When Cheddar cheese of different maturities was mixed with white soft cheese to prepare a CSS-free cheese emulsion, more extensive phase separation was observed compared to in their counterparts prepared with CSSs [34]. The level of phase separation increased with the age of Cheddar cheese, which was attributed to increased proteolysis during storage. Intact β-casein, which is amphiphilic, can act as an emulsifier. However, a decrease in intact β-casein with increased proteolysis reduces its emulsifying ability, reducing the emulsifying ability of aged Cheddar cheese [34]. Moreover, the loose structure, due to degraded protein, resulting from proteolysis contributes to a weakened protein network, causing reduced viscosity in cheese emulsions as the age of the cheese used in manufacturing increases [11]. The higher fat content (26–31%) and lower calcium content in Camembert cheese have been shown to result in better cheese emulsion stability by da Silva, Tziouri, Ipsen and Hougaard [35]. A lower calcium content increased the hydration of caseins, improving fat emulsification in the absence of CSSs [35].

Reducing fat content in dairy products is a challenge because fat plays a crucial role in the textural and rheological attributes of dairy products. Reduced-fat cheese emulsions have been shown to be more viscous than full-fat cheese emulsions at the same total solids level and can present pumping difficulties prior to drying [18]. This increase in viscosity is associated with higher protein content. The addition of microparticulated whey protein to cheese emulsion reduced the apparent viscosity and increased the flow behavior index, which consequently improved pumping ability and suitability for the drying of reduced-fat cheese emulsions [15]. Moreover, the reduced fat in cheese emulsions has been shown to lead to a denser and more compact network of proteins as observed in the microstructure. However, addition of microparticulated whey protein was shown to help forming a more porous structure, where fat globules were entrapped by the protein network [15]. The influence of various compositional and processing factors on the properties of cheese emulsion and cheese powder are summarized in Table 3.

### 4.2. Calcium-Sequestering Salts

To ensure the desired quality in cheese powder, the stability of cheese emulsion throughout processing is a crucial factor. The main ingredient in cheese emulsion is natural cheese, which has a calcium–para-casein network stabilizing the fat and water phases in the matrix. However, this network is disrupted during heating, leading to the separation of fat and water phases in the absence of CSSs. Therefore, phosphate- and citrate-based CSSs are used to adjust the pH and enhance the emulsification capacity of caseins through calcium sequestration [38]. CSSs play a crucial role in the emulsification of fat by the protein in the matrix, ensuring a uniform fat distribution in the cheese emulsion [6,7,39]. During industrial production of cheese powder, it is imperative to achieve a cheese emulsion without protein precipitation or creaming, ensuring homogeneity and pumpability of the feed. The colloidal stability and viscosity of the cheese emulsion, which are primarily influenced by the type of cheese, solids level, pH of the feed, processing variables, and CSSs, are considered as critical properties for powder preparation [5,9]. The absence of CSSs in cheese emulsions results in instability, phase separation, lumps, and sticky mass formation during heating [36].

### 4.3. Addition of Dairy Ingredients

Several studies have investigated the influence of the addition of various dairy ingredients on the properties of cheese emulsions with the aim of reducing CSSs in cheese powder. Both sodium caseinate and buttermilk powder exhibit good emulsification properties [5]. In emulsions of Cheddar cheese and soft white cheese, the addition of sodium caseinate (4%, *w*/*w*) increased the average particle size of the cheese emulsion and decreased the viscosity, whereas the addition of buttermilk powder (4% *w*/*w*) decreased the particle size and slightly increased the viscosity [5]. Cheese emulsions containing both sodium caseinate (2%, *w*/*w*) and buttermilk powder (2%, *w*/*w*) showed no visible cream layer separation or sedimentation after centrifugation, indicating a synergistic effect in fat emulsification [5]. Similarly, the addition of sodium caseinate and buttermilk powder (2% each) and buttermilk powder alone (4%) decreased the viscosity of Danbo and white cheese emulsions [11]. This decrease in viscosity was attributed to the reduced fat droplet size, owing to the emulsifying ability of buttermilk powder and sodium caseinate. No phase separation was observed in cheese emulsion with added sodium caseinate and buttermilk powder, indicating good colloidal stability and emulsification ability [11]. Therefore, the addition of sodium caseinate and buttermilk powder is a promising strategy for producing stable CSS-free cheese emulsions.

### 4.4. pH and Homogenization

Variations in the pH of cheese emulsions affect the functionality of caseins and the solubility of the calcium phosphate. Therefore, a few studies have investigated the effect of varying the pH of the cheese emulsions to circumvent the use of CSSs. Altay, Mendes, Sloth, and Mohammadifar [9] found that increasing pH from 5.1 to 6.8 decreased the solubility of colloidal calcium phosphate (CCP) from para-casein micelles in Irish Cheddar cheese emulsion, increasing fat emulsification and retention of fat and protein in the retentate of CSS-free cheese emulsions. The particle size and viscosity of cheese emulsion decreased with increasing pH from 5.1 up to pH 6.0, but a further increase in pH from 6.0 to 6.8 resulted in increased particle size and viscosity of the emulsion [9].

Another approach, based on acidification of the cheese emulsion to a pH of 4.2 or 4.7 followed by re-neutralization to pH 5.0 (similar to the control with pH 5.0), was investigated by the authors of [40] to understand the effect on CCP solubilization and the stability of emulsion. It was found that the solubilization of CCP was dependent on the level of pre-acidification. For instance, cheese emulsions acidified to pH 4.7 and re-adjusted to pH 5.0 had higher recovery of minerals and other components in cheese emulsion obtained after filtration than did the control without pH cycling, indicating that mineral solubilization was irreversible when compared to another sample (which was acidified to pH 4.2 and re-adjusted to pH 5.0). Despite differences in mineral solubilization, however, all the samples were unstable, showing creaming and sedimentation, making them unsuitable for drying [40].

The viscosity of cheese emulsion affects the foaming properties during processing, thereby impacting the amount of air retained in the cheese powder. This, in turn, can influence the oxidative stability and flavor retention of the final product [2]. Furthermore, excessively viscous feed can cause atomization problems during spray-drying, which can result in larger powder particles that dry slowly, leading to a darker-colored powder with a greasy texture and poor flowability [41]. High-fat CSS-free cheese emulsions prepared from cream cheese contained larger fat and protein aggregates, leading to higher viscosity than in control samples with CSSs [6]. Homogenization of a CSS-free cheese emulsion at 18 MPa reduced the particle size, while an increase in pH from 5.7 to 6.0 did not significantly affect emulsion stability. However, both pH adjustment and homogenization resulted in inferior-quality cheese emulsions compared to those made with added CSSs [6]. Overall, pH can play a major role in determining the stability of cheese emulsion, but alterations in pH alone do not appear to be a suitable method of replacing CSSs in cheese emulsions.

### 4.5. Ohmic Heating

Altay et al. [42] explored the use of ohmic heating to enhance mineral solubilization from Cheddar cheese in the preparation of a CSS-free cheese emulsion. Increased solubilization of calcium (three times higher), phosphorous, and protein (7% of total protein) from the cheese matrix were observed after ohmic heating compared to after conventional heating using water bath, presumably due to the electroporation effect induced by electric potential during ohmic heating. The solubilization effect on minerals was more pronounced at higher temperatures and holding times, while a negative correlation was observed with the voltage gradient. The increased conductivity with rising temperature reduced the viscosity and enhanced the ion mobility [42]. However, cheese emulsions prepared with ohmic treated cheese showed inferior colloidal stability in the absence of CSSs.

There is potential in exploring rapid heating technologies or non-thermal processing methods in cheese emulsion preparation. However, scalability might be a concern with these technologies [27]. Despite current research focusing on the use of various ingredients and alternatives for replacing CSSs in cheese emulsions, there is no method available to fully mimic the properties of CSSs [5]. As cheese powder produced with alternative techniques has inferior properties, the search is still ongoing for potential CSS replacement. Furthermore, natural ingredients such as egg yolk or lecithin could be explored to replace CSSs in cheese powder manufacture. Though it is a non-dairy ingredient, it could still aid in reducing sodium from cheese powder by replacing emulsifiers [15].

## 5. Drying of Cheese Emulsions

In commercial production, the drying of cheese emulsion is achieved through spray-drying, which can be as a single-stage or two-stage process. Two-stage spray-drying processes are the most commonly used in cheese powder production, where a belt or fluidized bed dryer can be employed as second drying stage after the main drying chamber in order to achieve a lower moisture content [8]. Another drying method studied for production of cheese powder is foam-mat drying, although, it is not used commercially yet.

### 5.1. Spray-Drying

Spray-drying is the most common drying technique used in cheese powder production. It is a type of continuous convective drying, where atomized feed droplets are dried by hot air inside the drying chamber [43]. Both a rotary disk atomizer and nozzle atomizer can be used for atomization of the cheese emulsion in cheese powder production. The drying parameters, such as air flow rate, atomizing speed, and inlet and outlet air temperatures, play a crucial role in determining properties such as the bulk density, solubility, wettability, color, and flavor of the cheese powder [1,44]. Erbay, Koca, Kaymak-Ertekin, and Ucuncu [44] evaluated the influence of spray-drying parameters on the characteristics of white cheese powder. Highly soluble and dispersible cheese powders have been obtained using a lab-scale spray-dryer at inlet air temperature, outlet air temperature, and atomization pressures of 174 °C, 68 °C, and 354 kPa, respectively. Higher outlet temperatures have been associated with increased browning and reduced solubility [44].

Free fat in cheese powder can cause flavor deterioration and impaired powder flow [2]. Free fat content was most affected by outlet air temperature and atomization pressure, which showed an inverse relationship with free fat content [44]. In a spray-drying study of concentrated milk, it was reported that a nozzle (pressure) atomizer was more effective in homogenization than a rotary atomizer [45]. Increased atomization pressure can produce effects similar to homogenization, leading to reduced particle size and increased surface area. This, in turn, allows higher surface coverage of fat by protein particles, thereby reducing free fat content, especially when combined with lower inlet and higher outlet air temperatures [44]. Similarly, Koca et al. [46] reported increased browning and decreased solubility of powder from white cheese with increasing outlet air temperature. Increased browning was attributed to the Maillard reaction upon heating. Cheese powders manufactured with the combination of sodium caseinate and buttermilk powder showed higher browning due to the higher lactose content in buttermilk powder, causing an increase in browning reactions [11].

Retention of free fatty acids (FFAs) in cheese powder is important for their function as a flavoring agent. FFA retention in Cheddar cheese powder was reported to be improved by addition of CSSs, which was attributed to reduced migration rate of fat droplets due to stabilization by the solubilized proteins. Moreover, increased atomization speed and cheese emulsion viscosity decreased retention of FFAs, whereas lower inlet temperatures promoted FFA retention. It was also suggested that reduction of free fat promotes the retention of volatile compounds [47].

The design of the spray-dryer producing cheese powder has a notable impact on the powder’s properties. The tall-form dryer integrated with a fluidized bed system is the most common in Europe, whereas filter-mat dryers are widely used in the USA [32]. Although single-stage dryers are not commonly used in cheese powder production, single-stage silo-form dryers, featuring drying towers (which are approximately six times taller than tall-form dryers and utilize dehumidified air), can be employed [8]. Research indicates that two-stage tall-form dryers offer superior flavor retention and produce larger particles with enhanced flowability [2]. Cheese powders from Camembert, Emmental, and Danbo cheese dried with a rotary disk atomizer in single-stage drying exhibited smaller powder particle sizes compared to a two-stage spray-dryer integrated with a belt dryer [8]. The comparison between a single-stage dryer with a rotary disk atomizer and a two-stage dryer with a nozzle atomizer and an integrated belt dryer for Camembert, Emmental, and Danbo cheese powder resulted in particle sizes of 41 vs. 67 μm; 36 vs. 42 μm; and 35 vs. 51 μm, respectively. For belt drying, the coalescence of fat globules in high-fat cheese powder and higher moisture at the end of spray-drying leads to the formation of larger agglomerates [8].

A lab-scale study reported the highest yield of EMC powder from white cheese with no lumps at an air inlet temperature of 150 °C and an air flow rate of 400 L/h [48]. Lower temperatures led to insufficient drying, causing particles to adhere to the dryer surface and form lumps. Conversely, temperatures higher than 150 °C resulted in particles with a wrinkled surface due to rapid moisture evaporation [48]. Similarly, Salum et al. [49] dried EMC at a 150 °C air inlet temperature, a 9.1 mL/min feed flow rate, and a 28.4 m^3^/h air flow rate. However, the yield of the EMC was only 43.1% due to the higher fat content of cheese powders, causing stickiness on the dryer wall and difficulties in recovering powder from the cyclone [49].

Production of extended cheese powder using whey protein and maltodextrin or lactose increased the powder yield to 63.2–67.4% as compared to control cheese powder without using these ingredients (47.3%). The best formulation was with addition of whey protein concentrate and lactose along with homogenization at 500 bar rather than at 1000 or 1500 bar [50]. Higher air inlet temperature and airflow rate, along with lower feed flow rate, produced cheese powders with lower surface free fat, increased wetting time, and reduced bulk density and solubility. The increase in wetting time (decreased wettability), despite the lower surface fat content, was associated with the higher impact of bulk density and porosity of powders on this characteristic. It is believed that wettability is negatively affected by an elevation in surface fat content; however, opposing results were obtained in this study. These results indicate that the effect of bulk density on wettability is greater than that of surface fat content. Furthermore, the yield of cheese powder was lower, mainly owing to its high fat content [51].

One study used an interesting approach to produce cheese flavor powder wherein milk was fermented using mold (*A. elegans*) fermentation for two days followed by enzymatic hydrolysis using proteases and lipases. This suspension, after completion of the fermentation and enzymatic process, was dried using spray-drying; the resultant powder had higher contents of flavoring compounds compared to commercial cheese powder [19].

### 5.2. Foam-Mat Drying

Foam-mat drying is an alternative drying technique that has been studied for production of cheese powder, aiming to preserve natural bioactive compounds by utilizing lower temperatures and shorter drying times. Foam mat-drying involves the creation of a foamed cheese emulsion using foaming agents, followed by drying using hot air on belts or tray dryers [52]. Foam-mat drying results in a highly porous powder with improved rehydration abilities [52]. In a study where whey protein concentrate (1%, 3%, and 5% *w*/*w*) was used as a foaming agent to produce white cheese powder, a drying temperature of 65 °C with a 3 mm thickness of foam on the tray yielded the best cheese powder reconstitution properties [52]. Motamedzadegan et al. [53] explored the use of xanthan gum (1% and 3% *w*/*w*) and albumin (3% *w*/*w*) as foaming agents to create foam for drying ricotta cheese produced from whey and milk mixtures at temperatures of 40, 55, or 70 °C. Samples with a higher proportion of whey had better solubility than powder with a higher milk proportion in the formulation used for preparing ricotta cheeses [53]. This indicates that foam-mat drying can be tailored to specific conditions and ingredients to achieve desirable properties in the resulting cheese powder.

## 6. Effect of Different Ingredients on Cheese Powder Properties

The reconstitution properties of cheese powders are a crucial aspect as they influence the functionality of the powder in its end-use applications. Various dairy and non-dairy ingredients (e.g., sodium caseinate, buttermilk powder, and maltodextrin) have been explored in CSS-free cheese powders. Additionally, studies have investigated the impact of using different cheeses with varying maturation levels on the reconstitution properties of the powders.

### 6.1. Effect of Dairy Ingredients

The powder prepared from cheese emulsions with added buttermilk powder and sodium caseinate powder resulted in lower free fat content, smaller particle size, higher wettability, and increased solubility [11,37]. The reduction in free fat contributes to decreased powder particle surface hydrophobicity, whereas the presence of lactose enhances particle surface hydrophilicity, thereby improving reconstitution properties. Similarly, cheese powders prepared with buttermilk powder exhibited better dispersibility and hydrophilicity due to higher lactose content [37]. Sweet whey powder is utilized as a filler in the food industry to enhance the reconstitution properties of powders. The addition of sweet whey powder to cheese emulsion prior to drying to replace 5% (*w*/*w*) cheese solids reduced the particle size and improved solubility during storage. This improvement was attributed to the hygroscopic nature of lactose present in sweet whey powder [10].

High fat content in some dairy powders poses challenges in reconstitution, such as reduced flowability, stickiness, and caking. Consequently, efforts have been made to produce reduced-fat cheese powder using microparticulated whey protein as a fat substitute [16]. Flowability, wettability, and dispersibility were enhanced in reduced-fat cheese powders and cheese powders with microparticulated whey protein compared to full-fat control cheese powders, as lowering the fat content reduces the hydrophobicity of powders, thereby improving these properties. However, lower solubility was found in reduced-fat cheese powders, as higher protein amounts lead to increased denaturation or aggregation of milk protein during thermal processing [16].

A study by da Silva, Tziouri, Ahrné, Bovet, Larsen, Ipsen and Hougaard [37] observed that cheese powder produced from cheeses with different ripening times (16, 30, and 45 weeks) exhibited differences in rehydration ability. As cheese ages, protein degradation occurs, leading to a loose structure of aged cheeses and decreased rehydration ability compared to younger cheeses. Moreover, powder made from 45-week-old cheese had the fastest dispersibility, yet total rehydration was best in the youngest cheese. Therefore, the maturity of cheese used in the manufacture of cheese powder affects the reconstitution properties of the powder. Cheese powder produced from 30-week-old cheese was found to have a narrower particle size distribution followed by 16-week-old cheese and 45-week-old cheese [11]. In the production of high-fat cheese powders, the role of homogenization and pH was investigated as an alternative to CSSs. While the investigated pH values (pH 5.7 and 6.0) did not result in differences, homogenization of cheese feed has been found to reduce the particle size of powder to one even lower than that achieved with CSSs [6]. Despite the differences in particle size, samples containing CSSs showed better reconstitution behavior. This improvement is associated with the enhanced casein solubilization, leading to increased rehydration ability in the presence of CSSs [6].

These investigations seek options to enhance the reconstitution properties of cheese powders. Understanding the contributions of different ingredients and cheese types to rehydration and fat emulsification in cheese powder is crucial for optimizing their functionality for various applications.

### 6.2. Effect of Non-Dairy Ingredients

Maltodextrin is commonly used as a filler in the food industry and is known for its ability to improve the bulk density and solubility of milk powders [12]. Maltodextrin addition led to the reduced viscosity of cheese emulsions and the free fat content of cheese powders, enhancing reconstitution properties in powder [12,14]. Turk-Gul, Urgu-Ozturk, and Koca [14] found that the addition of maltodextrin at 10% and 20% to replace cheese dry matter improved the reconstitution properties of cheese powder, as higher percentages led to fat separation and instability of emulsions. Similarly, the addition of maltodextrin at 30% by replacing cheese dry matter improved the wettability, dispersibility, and solubility of cheese powder and also resulted in a higher bulk density [12]. These improvements were associated with reduced viscosity of the cheese emulsion and reduced free fat content. The lower fat content in samples with added maltodextrin limited the formation of fat bridges between particles, contributing to improved wettability, dispersibility, and solubility [12,14]. Powders with added maltodextrin showed wrinkled surfaces and large, spherical structures. This is in contrast to samples without maltodextrin, which had smooth surfaces and agglomerated particles. This difference is likely due to the higher amount of fat on the surface of the samples with maltodextrin [12]. Additionally, cheese powder with maltodextrin showed a decrease in non-enzymatic browning in freshly produced powder, yet browning increased during storage [13]. The increase in browning was higher in samples with added maltodextrin compared to samples with added whey powder; samples with added whey powder showed increased browning due to the presence of lactose. The presence of dextrose in maltodextrin, along with its interaction with amines during storage, contributed to a greater increase in browning than lactose [13]. The addition of maltodextrin has been instrumental in improving the rehydration properties of cheese powder. However, it could potentially affect flavor or emulsifying abilities of the resultant powder in its end applications. Therefore, future studies are required to understand the effect of maltodextrin on cheese powder’s emulsifying abilities.

## 7. Changes in Cheese Powder during Storage

There are few studies published on the storage stability and shelf life of cheese powder. Some studies have reported the progression of non-enzymatic browning and caking tendency of cheese powder during storage. Kilic et al. [54] observed that non-enzymatic browning in cheese powder increases at higher water activity (A_w_) and higher temperature. The formation of initial browning products could have happened during manufacturing of cheese powder owing to thermal processing, leading to the progression of browning during storage. Further, the development of off flavors (cardboard-like, stale, oxidized) and a decrease in cheese flavor intensity has been also observed during storage. Cheese powder stored at A_w_ 0.54 at 20 °C was reported to have the longest shelf life and stability for 5 months as compared to powders stored at the same A_w_ at 30 and 40 °C [54]. A decrease in fat and lactose crystallinity and an increase in the moisture content of cheese powder was observed with increasing A_w_ (0.4, 0.5, 0.6 and 0.7) during storage at 21 °C. Cheese powder when compared with milk powder had lower crystallinity at all levels of relative humidity during storage, owing to presence of higher minerals in cheese powder than in milk powder. A higher mineral content contributed to increased water absorption and reduced crystallinity in cheese powder [55]. Generally, the lower the crystallinity, the higher the caking tendency; therefore, cheese powders have higher caking tendency than milk powders [55].

## 8. Conclusions

This review examined the applications and preparation methods of both cheese emulsions and cheese powders. Cheese powder has a wide range of applications as a flavoring ingredient, and its potential applications are expected to expand in the future due to its promising emulsification capacity. Furthermore, this review delves into the alternatives employed for replacing CSSs in producing clean-label cheese powders and discusses their impact on the properties of such powders. The ongoing quest for substitutes to replace CSSs in the formulation of cheese emulsion is emphasized, highlighting the need for comprehensive research in order to understand the structure–function relationship of potential ingredients that could replace CSSs in cheese powder production. There is a need for further studies on the effect of different CSSs on the properties of cheese powder. Non-thermal processing methods could also be explored for the preparation of cheese emulsion and improving the quality of cheese powder. In the future, cheese powder could be used as a flavor enhancer during the manufacture of cheese processed from young cheeses in order to produce a typical cheese flavor.

## Figures and Tables

**Table 2:** Processing conditions across references for preparation of cheese emulsion.

Dry Matter (% *w*/*w*)	Mixing Speed (rpm)	Heating Conditions	Calcium-Sequestering Salts	Reference
45	1500	85 °C for 45 s	Disodium phosphate (4%, *w*/*w*)	[5]
38	-	80 °C for 5 min	Disodium phosphate (3.5%, *w*/*w*)	[6]
35	1000	85 °C for 20 min	-	[7,9]
36–38	-	-	Disodium phosphate (3.5%, *w*/*w*)	[8]
25	6000	80 °C for 10 min	Joha S-85 (3%, *w*/*w*)	[12,13]
25	6000	80 °C for 10 min	Joha (3%, *w*/*w*)	[33]
36	1500	80 °C for 1 min	-	[35]
38	1500	87 °C for 45 s	Disodium phosphate	[36]
-	1500	87 °C for 45 s	Disodium phosphate (2.5%, *w*/*w*)	[34]

**Table 3:** Effect of different compositional and processing factors on the properties of cheese emulsion and cheese powder.

Factors	Effect on Cheese Emulsion	Effect on Cheese Powder	References
Cheese maturity	Mature cheese: high flavor intensity and reduced viscosity Young cheese: higher intact casein and difficult to dissolve	Powder made from old cheese has higher dispersibility and narrower particle size distribution	[37]
Fat content	Lower fat content provides a compact protein network and higher viscosity, causing pumping difficulties	High fat content reduces powder solubility and causes stickiness and caking	[15,16,18]
Reduced CSS content	Protein aggregates and irregular fat distribution without CSSs	Low powder recovery	[7,34]
pH	pH increase above 5.1 enhances fat emulsification, viscosity and fat retention	pH 5.7 and 6.0 cheese emulsion showed no variation in cheese powder characteristics	[6,9]
Homogenization	Reduced particle size and facilitates atomization	Reduced particle size of powder	[6]
Addition of sodium caseinate and buttermilk powder	Improved emulsification and reduced viscosity	Lower free fat content, smaller particle size, higher wettability, and increased solubility	[5,11,37]

## Data Availability

The original contributions presented in the study are included in the article; further inquiries can be directed to the corresponding author.
